# The influence of fire frequency on the structure and botanical composition of savanna ecosystems

**DOI:** 10.1002/ece3.5400

**Published:** 2019-07-02

**Authors:** Natasha Ribeiro, Gernot Ruecker, Navashni Govender, Valério Macandza, Aurélio Pais, Domingos Machava, Aniceto Chauque, Sa Nogueira Lisboa, Romana Bandeira

**Affiliations:** ^1^ Faculty of Agronomy and Forest Engineering Eduardo Mondlane University Maputo Mozambique; ^2^ ZEBRIS GbR München Germany; ^3^ Conservation Management Kruger National Park Skukuza South Africa; ^4^ Nelson Mandela Metropolitan University George South Africa

**Keywords:** African Savannas, biodiversity, fire ecology, protected areas

## Abstract

Savannas cover 60% of the land surface in Southern Africa, with fires and herbivory playing a key role in their ecology. The Limpopo National Park (LNP) is a 10,000 km^2^ conservation area in southern Mozambique and key to protecting savannas in the region. Fire is an important factor in LNP's landscapes, but little is known about its role in the park's ecology. In this study, we explored the interaction between fire frequency (FF), landscape type, and vegetation. To assess the FF, we analyzed ten years of the Moderate resolution Imaging Spectroradiometer (MODIS) burned area product (2003–2013). A stratified random sampling approach was used to assess biodiversity across three dominant landscapes (Nwambia Sandveld‐NS, Lebombo North‐LN, and Shrubveld Mopane on Calcrete‐C) and two FF levels (*low*—twice or less; and *high*—3 times or more, during 10 years). Six ha were sampled in each stratum, except for the LN versus high FF in which low accessibility allowed only 3 ha sampling. FF was higher in NS and LN landscapes, where 25% and 34% of the area, respectively, burned more than three times in 10 years. The landscape type was the main determinant of grass composition and biomass. However, in the sandy NS biomass was higher under high FF. The three landscapes supported three different tree/shrub communities, but FF resulted in compositional variations in NS and LN. Fire frequency had no marked influence on woody structural parameters (height, density, and phytomass). We concluded that the savannas in LNP are mainly driven by landscape type (geology), but FF may impose specific modifications. We recommend a fire laissez‐faire management system for most of the park and a long‐term monitoring system of vegetation to address vegetation changes related to fire. Fire management should be coordinated with the neighboring Kruger National Park, given its long history of fire management. *Synthesis*: This study revealed that grass and tree/shrub density, biomass, and composition in LNP are determined by the landscape type, but FF determines some important modifications. We conclude that at the current levels FF is not dramatically affecting the savanna ecosystem in the LNP (Figure 1). However, an increase in FF may drive key ecosystem changes in grass biomass and tree/shrub species composition, height, phytomass, and density.

## 1 INTRODUCTION

Savannas constitute one of the largest biomes in the world, covering about 20% of the land surface (Scholes & Hall, 1996). Africa holds most of the world's savannas area (Shorrocks, 2007), with about 54% of the surface covered by these ecosystems (Cowling, Richardson, & Pierce, 1997). In sub‐Saharan Africa, savannas occupy 60% of the land surface and their distribution and structure is determined largely by rainfall, nutrient availability in the soil, geology (defining the landscape type, Stalmans, Gertenbach, & Carvalho‐Serfontein, 2004), herbivory, and fire (Scholes & Walker, 1993).

Known as the “Fire Continent”, Africa supports widespread biomass burning (Trollope & Trollope, 1997), which is recognized as one of the most important disturbances impacting ecosystem processes. There is much evidence that fire has an important role in maintaining the composition, structure, and function of African ecosystems (Frost, 1984, 1985). Fire is also one of the key factors in maintaining the competitive balance between trees and grasses in savanna (Higgins et al., 2007; Higgins, Bond, & Trollope, 2000; Trollope & Trollope, 1997). Many plant species have adapted to fire regimes and have growth and reproductive attributes linked to the local fire cycles. Similar examples can be found for birds and mammals, which suggest that fire has a long evolutionary history in African savannas (Bendell, 1974; Gregory, Sensenig, & Wilcove, 2010; Trollope, 1984).

The historical fire regimes, including their natural variability in time and space, are not extensively known in the region, albeit this knowledge would be important for fire management (Archibald, Scholes, Roy, Roberts, & Boschetti, 2010). Fire management, like other forms of ecosystem management, needs to be continually adaptive in order to support decisions based on environmental and societal changes and as knowledge improves (van Wilgen & Biggs, 2011). Therefore, studying the relationship between fire and the ecology of savannas is important to understand key ecosystems processes, biodiversity, and support decision‐making.

The aim of this study was to explore the relationships between fire frequency, landscape, vegetation structure, and botanical composition of the savannas in the Limpopo National Park (LNP), southern Mozambique. We defined two research questions for this study:

1. Does fire frequency influence grass biomass and botanical composition in different landscape types?
2. Does fire frequency influence the height, biomass, and botanical composition of woody plants in varied landscapes?

Despite the short‐term analysis of the fire regimes in this study (10 years, 2003‐2013), our analyses provide insights of the interactions among the main drivers of vegetation structure and composition in the LNP.

## 2 MATERIALS AND METHODS

### 2.1 Study area

This study was carried out in the Limpopo National Park (LNP; 10,000 km^2^), southern Mozambique. The park is part of the Great Limpopo Transfrontier Park (GLTP), which is a 35,000 km^2^ conservation area that also includes the Kruger National Park (KNP) in South Africa and the Gonarezhou National Park (GNP) in Zimbabwe (Figure 2).

**Figure 1:**
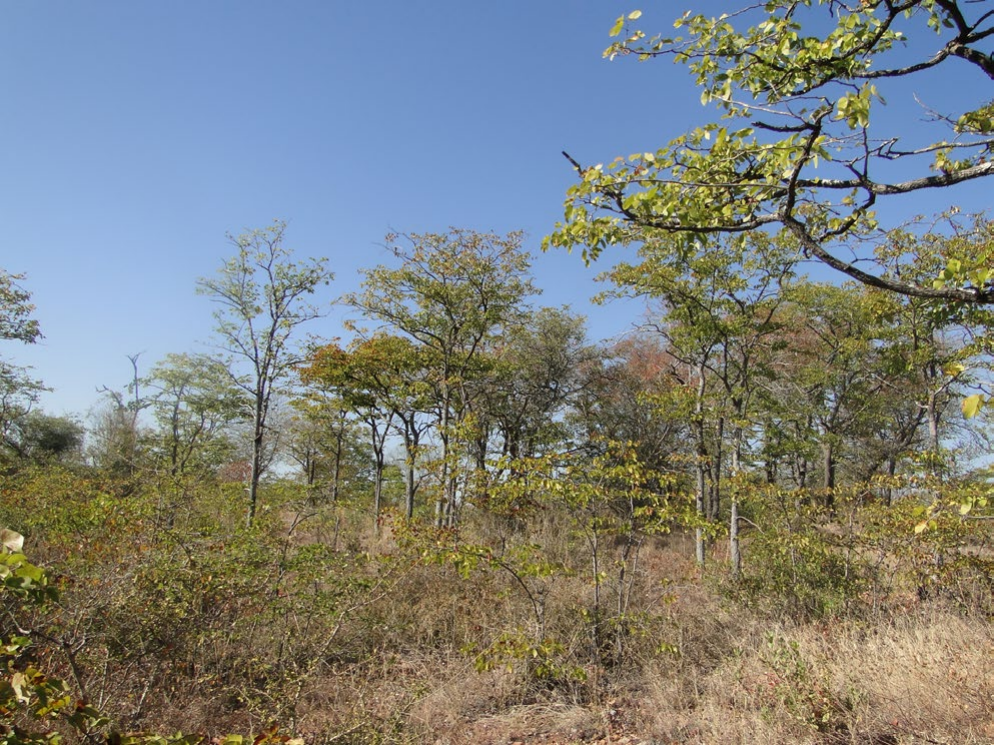
Illustration of a typical mopane savanna in Limpopo National Park (Photo credits: N. Ribeiro)

**Figure 2:**
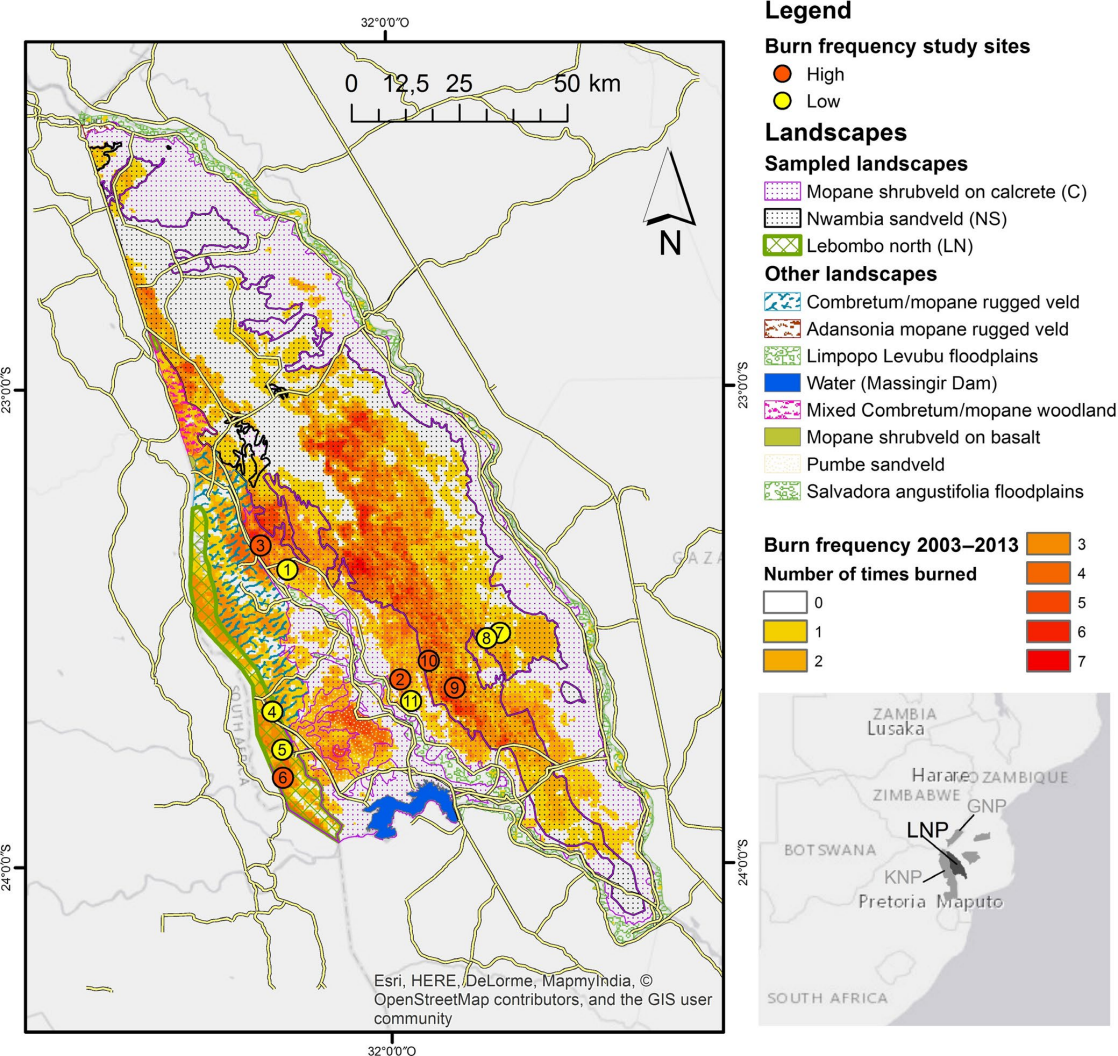
Location of sampling sites according to the landscape type and fire frequency (2003–2013). Inset map: location of LNP within the Great Limpopo Transfrontier Park (KNP: Kruger National Park; GNP: Gonarezhou National Park)

The climate in LNP is classified as warm dry tropical with mean annual precipitation increasing from 360 mm to over 500 mm from North to South. Rainfall is concentrated in the months of November to April. Mean annual temperatures fluctuate between 24°C and 30°C (ANAC, 2003). Altitude in the park varies between 260 and 840 m above sea level. The southern region of the LNP is dominated by rhyolite volcanic rock, while the North consists of red sand mantle, whereas alluvium and clay sediments and calcaric sedimentary rocks characterize the Limpopo floodplains.

Vegetation variations associated with geologic conditions within the landscapes are observed in the park. Fifteen landscapes types were identified and described by Stalmans et al. (2004). The study presented here was carried out in three dominant landscapes: Nwambia Sandveld (NS; 44% of the LNP's total area), Lebombo North (LN; 3.5% of LNP along the western boundary with KNP), and mopane shrubveld on Calcrete (C; 38.8% of LNP). NS is found in sandy substrates, including deep red soils. LN is extremely stony with shallow soils derived from rhyolites and C occupies the sedimentary footslopes and ravines with calcareous pebble‐beds, with shallow and calcareous soils.

LNP support a variety of large grazing and browsing mammal species, notably elephant (*Loxodonta africana*), buffalo (*Syncerus caffer*), giraffe (*Giraffa camelopardalis*), zebra (*Equus burchelli*), impala (*Aepyceros melampus*), and blue wildebeest (*Connochates taurinus*).

About 25,000 people live within the park limits and use fire to promote hunting and agriculture. Lack of scientific records limits our understanding about the exact causes of fires as well as the relative contribution of natural and anthropogenic fires. Since the park has no fire management system in place, it is safe to affirm that fires in the LNP are stochastic.

### 2.2 Analysis of the fire frequency from remotely sensed data

We analyzed fire frequency (FF) from 2003 through 2013 using data from the Moderate Resolution Imaging Spectroradiometer (MODIS) sensor on board of NASA's Terra and Aqua satellites. We obtained the collection 6 MODIS burned area product called MCD64 (Giglio, Loboda, Roy, Quayle, & Justice, 2009; Giglio, Boschetti, Roy, Humber, & Justice, 2018) from the University of Maryland, and the collection 6 MODIS active fire product (called MOD14 for Terra/MYD14 for the Aqua satellite) from NASA (Giglio, Schroeder, & Justice, 2016; Justice et al., 2002). In accordance with Archibald et al. (2010), we defined a fire event as a group of burned area pixels that are adjacent in both time and space (i.e., pixels touch in space and are no more than two days apart) and devised an algorithm that identifies fire events based on these criteria by searching through all datasets. For analyzing the fire history of the selected sampling sites, we checked both the burned area and active fire products for fire occurrences during the observation period.

### 2.3 Field sampling approach

**Figure 3:**
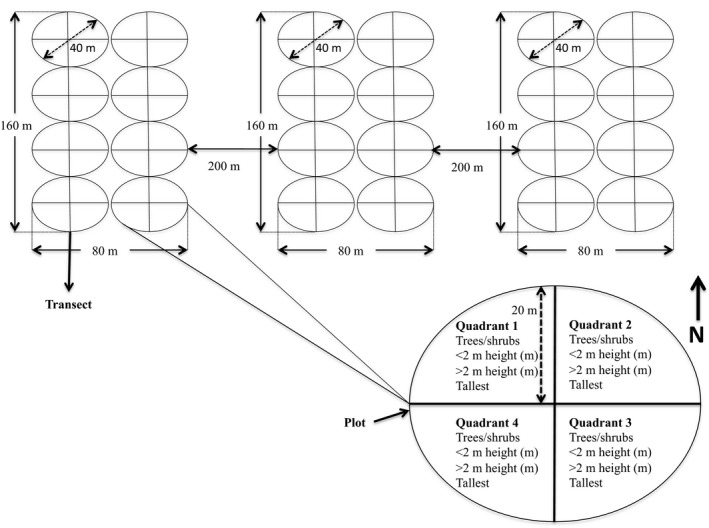
Layout of the sampling site to assess vegetation. In Nwambia sandveld and Calcrete landscapes four (two in low FF and two in high FF) were allocated and in Lebombo North three (one in high FF and two in low FF) were allocated

In this study, we used a stratified random sampling approach (Thompson, 2012) using landscape and FF as strata. Sites were categorized as high or low fire frequency if they burnt more or less than three times, respectively, between 2003 and 2013. Within each stratum, we randomly selected sampling sites (Figure 3): four (two in low FF and two in high FF) in each NS and C landscapes and three (one in high FF and two in low FF) in the LN. The latter occurs in a narrow strip along the border between LNP and KNP (Figure 2), which limited our chances of getting a second accessible site in the high FF stratum. For all sampling sites, we analyzed fire history in more detail establishing the most probable burn dates from the remotely sensed data.

The Adapted form of the Point Centre Quarter (APCQ) technique developed and described by Cottam and Curtis (1956) was used for conducting vegetation surveys. APCQ is comprised of 8 circular plots of 40 m in diameter each, arranged in two contiguous parallel transects (i.e., 4 plots per transect). In each sampling site, we established 3 replicates of those adjacent transects (Figure 3), totaling 48 plots (24 plots for LN vs. high FF) or a sampling effort of about 6 ha (3 ha for LN vs. high FF) per landscape type.

### 2.4 Data collection

#### 2.4.1 Tree/shrub vegetation

Within each plot the following parameters were measured for the nearest rooted tree/shrub in each of the four quarters surrounding the central recording point: viz. species (Figure 3): (a) the tree/shrub botanical species name; (b) base diameter (cm) at ground level for individuals ≤2 m high; (c) diameter at the breast height (dbh; cm) for individuals >2 m high; and (d) overall height (m).

### 2.5 Grass vegetation

Grass biomass (of all species at a reading point) was estimated with a Disc Pasture Meter using 100 readings recorded at 3 meter intervals along the 2 plot transects (50 readings/transect; Trollope, 1990). In this study, we used the calibration relationships defined for the KNP by Trollope and Potgieter (1986). All grasses at the reading point were also identified by their botanical species names and the species tufts counted.

### 2.6 Data analysis

#### 2.6.1 Tree/shrub vegetation

For trees and shrubs, the following ecological parameters were estimated: (a) density (number trees or shrubs/ha); (b) phytomass [tree equivalents (TE/ha), TE = number of trees/shrubs of 1.5 m high; Trollope, Trollope, & Hartnett, 2002]; and (c) average height class: trees/shrubs ≤2 m (i.e., in the fire trap) and >2 m high (i.e., resprouting) and the tallest tree/shrub (i.e., out of the fire trap). For all plots, the tallest individual was always taller than 2 m.

Data analysis was performed in R software R (R Development Core Team, 2014) by using *glmmPQL* and *glmmML* packages (Bates, Mächler, Bolker, & Walker, 2015; Zuur, Ieno, Walker, Saveliev, & Smith, 2009). After testing for normality by using the Shapiro–Wilk test we detected that, our data violated the normality assumption for data distribution (*p* > 0.05). Thus, we applied a generalized linear mixed model (GLMM; Bolker et al., 2009) to address the influence of FF on woody density, average height, and phytomass. In our analysis, the FF was considered as the fixed effect (categorical analysis) and landscape type as a random determinant. The *F*‐test at 95% confidence interval was used to determine the influence of FF on woody density, average height, and phytomass.

The Importance Value Index (IVI; Kent, 2012) was determined for trees/shrubs, as per the formula:

$$\begin{array}{cc} \text{IVI} & =\text{Relative abundance in}\;\%\;\left(n_{i}/n_{t}\;*100\right)\\ & \;+\text{Relative dominance in}\;\%\;\left(g_{i}/G*100\right)\\ & \;+\text{Relative Frequency in}\;\%\;\left(f_{i}/f_{t}*100\right) \end{array}$$

where *n_i_* = abundance of species *i* (*n*/ha); *n_t_* = abundance of all species (*n*/ha); *g_i_* = basal area of species i (m^2^/ha); *G* = basal area of all species (m^2^/ha); *f_i_* = frequency of species *i* (number of plots in which the species occurred/ total number of plots); and *f_t_* = frequency of all species. By aggregating three important ecological parameters, this index provides a good measure of botanical composition and is an indication of which species are ecologically more important in the area under respective environmental conditions.

### 2.7 Grass vegetation

For grasses we calculated (a) average biomass; and (b) relative frequency of grass species responses to grazing pressure. The latter was determined by assigning grasses to one of the three categories in terms of their response to grazing pressure. *Decreaser* species decrease in abundance when rangeland is under‐or overgrazed, *Increaser I* species increase in abundance when rangeland is under‐ and/or selectively grazed, and *Increaser II* increase in abundance when rangeland is overgrazed (Dyksterhuis, 1949; Trollope, 1990). The relative frequency of these grass species categories was calculated for each landscape and FF.

After testing for normality by using the Shapiro–Wilk test, we detected that our data followed the normality assumption for data distribution (*p* < 0.05). A *t* test was performed to analyze the differences, at 95% confidence interval, in grass biomass response variable according to FF within a landscape.

A multi‐dimensional scaling (MDS) analysis was used to look at the response of grass species composition in relation to the three landscapes and FF. MDS is an ordination analysis, which permits investigation of relationships among a set of response and independent variables aiming at reducing data complexity for meaningful interpretation (Fenton & Pearce, 1988).

## 3 RESULTS

### 3.1 Description of the fire regime

**Figure 4:**
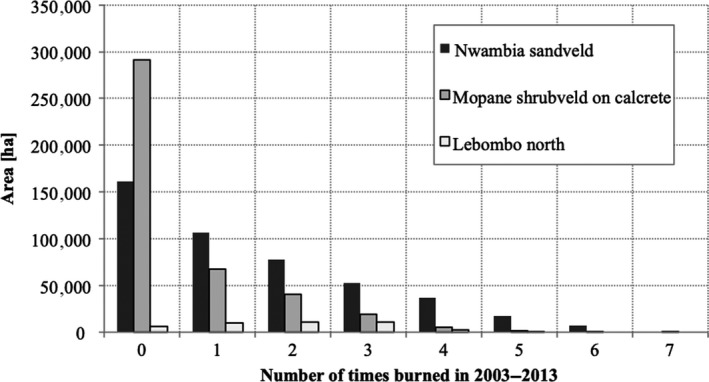
Area contribution (ha) of different burn frequencies (number of times burned in the observation period 2003–2013)

For the period of study (2003–2013), the burned area did not exceed 150,000 ha per year (15% of the total area of the park), except for 2004 when 350,000 ha (or 35% of LNP) were burned. The burned area showed a quasi‐biennial pattern, with largest area burned in 2004, 2006, and 2008. Fire frequency was higher in the NS and LN landscapes, where 25% and 34% of the area, respectively, burned more than three times in the observation period of 10 years (Figure 4), whereas only 6% of the area burned more than three times in C.

Inevitably, fire counts and hence frequency estimates are influenced by the inherent uncertainties of the burned area dataset, which have not been quantified for this landscape. For the Southern part of neighboring Kruger National Park (KNP) in South Africa, such an assessment has been done by Tsela et al. (2014) through comparison with higher resolution Landsat data using a previous version of the MODIS MCD64 burned area dataset (Giglio et al., 2009). In KNP, these authors found that omission error (i.e., burned area mapped as unburned) was between 34% and 25% while commission error (unburned mapped as burned) was lower (17 to 24%). If this is true also for the collection 6 dataset, it is expected that the true fire frequencies may be higher for the landscapes in LNP, although the collection 6 dataset is reported to be an improvement in terms of accuracy (Giglio et al., 2018).

**Figure 5:**
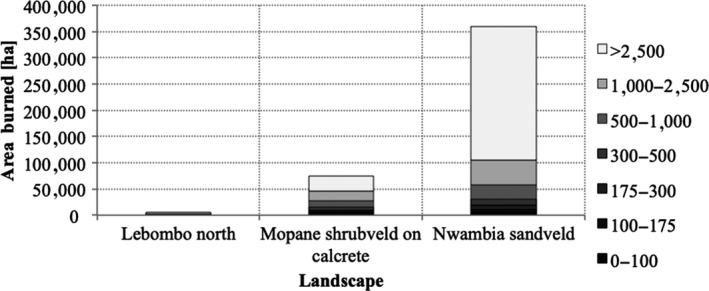
Area contribution (ha) of each fire size classes (ha) to total burned area 2003 through 2013 in the different landscapes of LNP

**Figure 6:**
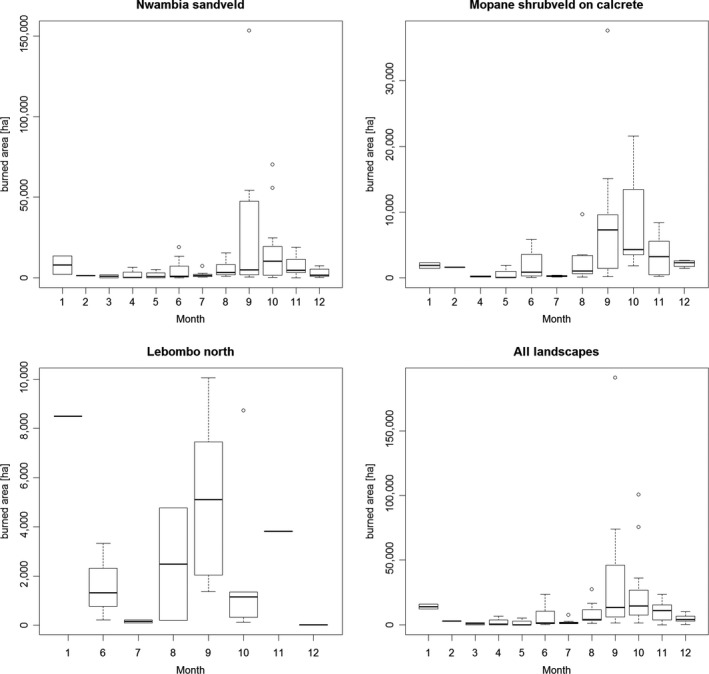
Boxplots of monthly burned area in the different landscapes based on the data from 2003–2013 (Nwambia Sandveld: NS; Mopane Shrubveld on Calcrete: C and Lebombo North: LN)

Large fires contributed the most to the burned area, with fires above 10,000 ha contributing more than 50% of the annual burned area. Fires on the C and in LN landscapes tend to be smaller than those occurring in the NS (Figure 5). Most area burned during the dry season mainly in August through November. This was more pronounced for years with a large area burned (2004, 2006, 2008, 2011, and 2013). There was substantial inter‐annual variability in the total monthly burned area (Figure 6).

### 3.2 Grass composition, biomass, and response to grazing pressure

**Figure 7:**
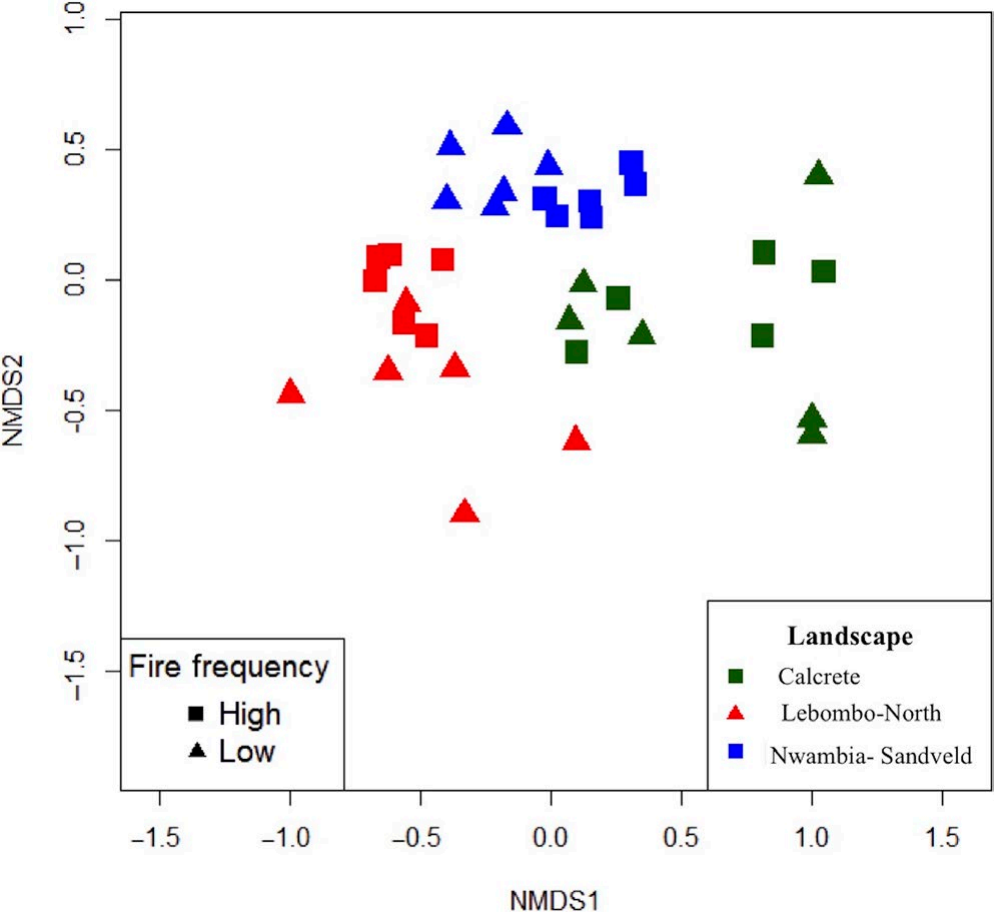
Multi‐Dimensional Scaling scatter plot of grass species composition grouped by (a) landscape and (b) fire frequency (NMDS1 and NMDS2 are the two first axes of the MSD analysis)

Species richness was higher (44 species) in Lebombo North (LN) and Calcrete (C), and somewhat lower (35 species) in Nwambia Sandveld (NS). Fire frequency did not affect grass richness (number of species) in all landscapes, and the MDS scatter plot (Figure 7) indicates that the landscape rather than FF drove botanical composition. However, within each landscape we found interesting relationships with FF. LN was dominated by *Heteropogon sp*., *Digitaria *sp., and *Pogonarthria squarrosa* (all *increaser I*) in low FF sites, while *Urochloa mossambicensis (increaser II)* stood out in high FF places. All species were present in sites with low and high FF, but differed in their densities. NS was dominated by *Digitaria eriantha* (*decreaser*) and *U. mossambicensis (increaser II)* in low FF sites and *D. eriantha* was still dominant in high FF places, but *U. mossambicensis* was not present. The C landscape was dominated by *D. eriantha, Penisetum glaucum,* and *Panicum maximum (decreaser)* in low FF sites, but when the FF increased *D. eriantha* (*decreaser*) became the dominant species.

**Figure 8:**
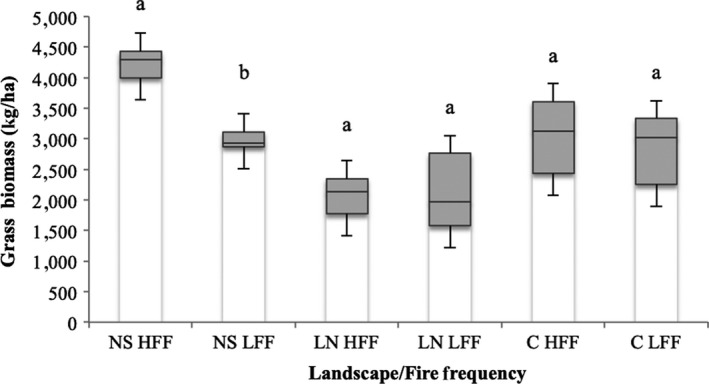
Box plot of average biomass (kg/ha) grouped by landscape and fire frequency (legend: NS, Nwambia Sandveld; LN, Lebombo North; C, Calcrete; HFF, High Fire Frequency; LFF, Low Fire Frequency; equal letters indicate non‐significant differences between FF within a landscape, at 95% confidence level)

Except for NS under high FF [average biomass in low FF = 3,046 kg/ha (±263.69) and average biomass in high FF = 4,215 kg/ha (±333.56); *p* < 0.05], grass biomass did not respond differently to landscape type and FF (Figure 8). The C showed the second highest grass biomass [average biomass in low FF = 2,881 kg/ha (±547.18 and average biomass in high FF = 3,055 kg/ha (±697.08); *p* > 0.05], while the rhyolite LN landscape had the lowest biomass [average biomass in low FF = 2,058 kg/ha (±761.21) and average biomass in high FF = 2,095 kg/ha (±378.45); *p* > 0.05].

### 3.3 Tree/shrub composition, density, height, and phytomass

The three landscapes in LNP were characterized by distinctive plant communities as shown in Table 1. NS corresponded to the *Combretum apiculatum* (IVI = 110) dominated tree savannas, but *Sclerocarya birrea* (IVI = 53) and *Xeroderris stuhlmannii* (IVI = 34) were also ecologically important. In this landscape, high FF promoted species such as *Combretum hereroense* (IVI = 34) and *Lannea schweinfurthii* (IVI = 46). The LN was dominated by *Colophospermum mopane* (IVI = 110) and *C. apiculatum* (IVI = 80) communities in both high and low FF areas, but in frequently burned sites *C. apiculatum* (IVI = 120) seemed to dominate over *C. mopane* (IVI = 98). The C landscape was almost homogeneous in terms of species composition, shrubby *C. mopane* the overwhelmingly dominating species, in both high (IVI = 200) and low (IVI = 160) FF situations.

**Table 1:** Importance Value Index (IVI) of dominant tree/shrub species per landscape and fire frequency in LNP

Species name	Importance Value Index (IVI)					
	Nwambia Sandveld (NS)		Lebombo North (LN)		Calcrete (C)	
	Low fire frequency	High fire frequency	Low fire frequency	High fire frequency	High fire frequency	Low fire frequency
*Combretum apiculatum*	110	123	86	123	12	18
*Sclerocarya birrea*	53	25	10	5	–	–
*Xeroderris sthulmannii*	34	27	–	–	–	–
*Senegalia nigrescensis*	26	–	–	–	21	13
*Grewia bicolor*	21	5	–	–		
*Lannea schweinfurthii*	20	46	11	6	–	–
*Combretum hereroense*	–	34			–	–
*Colophospermum mopane*	–	–	112	97	–	–
*Dialium *sp.	–	–	19	22	–	–
*Colophospermum mopane (shrubby mopane*)	–	–	–	–	165	203
*Euclea divinorum*	–	–	–	–	25	–
*Gymnosporia heterophylla*	–	–	–	–	13	2
*Terminalia sericea*	–	–	–	–	12	–
*Spirostachys africana*	–	–	–	–	10	–
*Dalbergia melanoxylon*	–	–	–	–	–	40

Note

High fire frequency = burned 3‐6 times and low fire frequency = burned <3 times, between 2003 and 2013.

Overall, tree/shrub phytomass, height, and density were not influenced by FF (*p* > 0.05), but they were significantly different among the three height categories (≤2 m, >2 m and tallest). Fire frequency did not influence any of the structural parameters in any height class (Table 2).

**Table 2:** Generalized mixed linear model (GMLM) of tree/shrub plants per landscape and fire frequency

Parameters	Variables	Value	*SE*	*t* test	*p*‐value
Effect of fire frequency					
Average height	High versus Low	−0.07	0.18	−0.39	0.6951
Tree/shrub density	High versus Low	0.14	0.13	1.13	0.258
Phytomass	High versus Low	290.75	242.38	1.2	0.2307
Effect of tree/shrub height class					
Average height	≤2 m	–	–	–	Non‐significant
	>2 m	1.69	0.18	9.46	<0.001
	Tallest	4.29	0.17	24.5	<0.001
Density	≤2 m	–	–	–	Non‐significant
	>2 m	−1.91	0.25	−7.63	<0.001
	Tallest	−2.77	0.36	−7.76	<0.001
Phytomass	≤2 m	–	–	–	Non‐significant
	>2 m	−1,643	239.29	−6.87	<0.001
	Tallest	−1,805.27	234.59	−7.7	<0.001
Interactive effect of fire frequency and tree height class					
Average height	Low FF: >2 m	0.11	0.25	0.44	0.663
	Low FF: tallest	0.38	0.25	1.54	0.12
Density	Low FF: >2 m	0.55	0.31	1.75	0.0798
	Low: tallest	−0.35	0.52	−0.68	0.5
Phytomass	Low: >2 m	−10.54	338.42	−0.031	0.9752
	Low: tallest	−313.11	329.38	−0.95	0.3421

**Figure 9:**
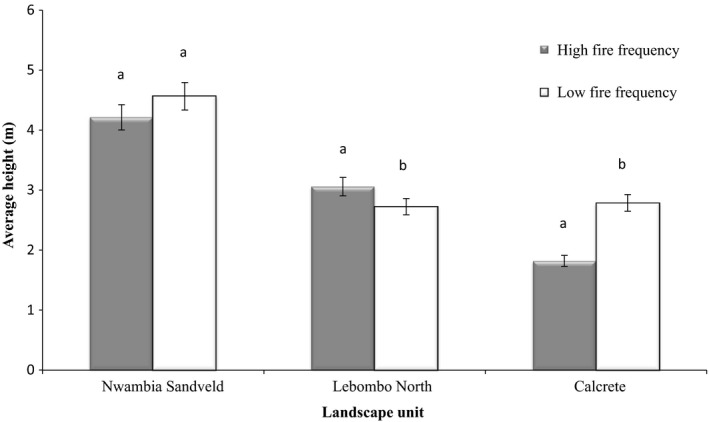
95% confidence interval of average height grouped by landscape and fire frequency (equal letters indicate non‐significant differences between FF within a landscape, at 95% confidence level)

Average height was significantly higher (average height = 4.0–4.5m; *p* < 0.01) in NS than in LN (average height = 2.7–3.1m) and C (average height = 1.8–2.7 m). However, the influence of FF varied according to landscape. In LN, high FF promoted taller trees, while the reverse happened in C and in NS height was not affected by FF (Figure 9).

## 4 DISCUSSION

### 4.1 Influence of fire frequency and landscape on grass botanical composition, biomass, and response to grazing pressure

In this study, the NS landscape presented higher grass biomass than the C and LN landscapes, which is probably a response to red sandy soils in the former. These are known to have better nutrient condition than the rocky soils in C and LN (Jones, Smithers, Scholes, & Scholes, 1990). Fire frequency had an influence on grass biomass only on sandy NS. In the LN and C landscapes, the high soil rockiness condition might have been responsible for breaking fire continuity thus reducing its effect on grass biomass. These results were similar to Buis et al. (2009) who found that annual fires stimulated total Annual Net Primary Production (ANPP; 28%–100%) relative to unburned treatments at sites with deep sandy soils, and FF had no effect on ANPP in sites with shallow rocky soils.

The landscape type was the main driver of the grass botanical composition and response to grazing pressure while FF had no influence. This is opposite to what was found in a recent analysis of a 50‐year‐old fire experiment in KNP in which annual burning resulted in the deterioration of the range condition in low‐rainfall mopane sites (bordering with the LNP; Trollope, 2004; Trollope, Wilgen, Trollope, Govender, & Potgieter, 2014). These apparently contradictory results may be an effect of stochastic occurrence of fire in LNP associated with the absence of formal management activities as opposed to KNP with a long history of regular fires.

Several studies in the region have demonstrated that heterogeneous frequent fires improve and maintain the nutritional quality of grasslands making it highly attractive to grazing animals (Aranibar et al., 2003; Munthali & Banda, 1992; Parsons, Shackleton, & Scholes, 1997; Shackleton, 1992; Trollope et al., 2014; West, 1965). Even though this hypothesis was not tested in our study, we consider that it is important to address it in the future, in view of establishing a fire management system with a focus on LNP's wildlife.

### 4.2 Influence of fire frequency and landscape on tree/shrub composition, height and phytomass

The three landscapes in LNP supported three different plant communities as described by Stalmans et al. (2004): NS landscape was a *Combretum apiculatum/ Xeroderris stuhlmannii* dominated savanna, shrubveld mopane dominated the C landscape and the rocky LN was characterized as *Colophospermum mopane*/*C. apiculatum* plant community. Fire frequency affected woody composition only in NS and LN landscapes. In the former high FF promoted species such as *Combretum hereroense* and *Lannea schweinfurthii,* while in high FF sites of LN *C. apiculatum* dominated over *C. mopane*. These observations may be an indication of species composition change in these ecosystems as a result of FF. However, these short‐term observations from this study are not enough to produce a conclusion and this should be a matter of long‐term monitoring.

Of the three landscapes studied, NS presented the highest average height of 4.0–4.5 m, followed by LN with 2.7–3.1 m and C with 1.8–2.7 m. Taller trees in NS are expected given the presence of deep, red sandy soils that allow trees to exploit resources in inner soil layers (Strydom, Rowe, Riddell, Govender, & Lorentz, 2014). The shrubby condition of *C. mopane* in C landscape is a result of rocky, calcareous soils conditions (Stalmans et al., 2004). Fire frequency did not affect tree/shrub overall mean height for all three landscapes. However, several classes were influenced differently in all landscapes. Low FF stimulated taller resprouting (*h* > 2 m) and adult trees (tallest). Additionally, in the C landscape high FF resulted in lower height of all trees/shrubs. These results can be explained by the fact that low FF allows small trees (equal or lower than 2 m high) to escape the fire trap (i.e., a cycle of biomass loss followed by resprouting, maintained by frequent fires, Werner & Prior, 2013) and reach the canopy level (Trollope, 1999). In addition, in the NS landscape low FF sites were associated to lower grass stocks and consequent reduced competition for light and nutrient thus, allowing woody species to grow out of the fire trap. According to several authors (Higgins et al., 2007, 2000; Ojeda, Brun, & Vergara, 2005; Trollope & Trollope, 1999; Trollope et al., 2002) once trees/shrubs have escaped the flame zone (*h* > 2 m) they are adapted to, and relatively unperturbed by frequent surface fires characteristic of savanna ecosystem. Moreover, tall tree/shrubs are able to benefit from the additional resources available on soils after a fire (Hanan, Sea, Dangelmayr, & Govender, 2008).

Tree density and phytomass did not differ among the three landscapes, but FF produced different responses per height class. Low FF resulted in increased tree/shrub density and phytomass in all height categories, similar to other studies in the region (Buitenwerf, Bond, Stevens, & Trollope, 2012; Enslin, Potgieter, Biggs, & Biggs, 2000; Trollope, 1998; Trollope, Trollope, Biggs, Pienaar, & Potgieter, 1998).

### 4.3 Implications for fire management

Human activities and natural factors are the main causes of fires in LNP and there are no regular fire management activities. Given that there are no records about the causes of fire in the park, it is impossible to discern the proportional contribution of each cause of fires occurring in our study area.

Based on our results, the current fire regime is not dramatically affecting the grass and woody vegetation in LNP, but there are specific responses that need to be taken into consideration in establishing a fire management system. For instance, systematic annual burning in NS may result in higher grass biomass stocks, which in turn may reduce tree/shrub height fire trap. The latter is also expected for the C landscape, while in the LN systematic frequent burnings may continuously topkill tree/shrub in the fire trap, reducing density. In the latter, a change in tree species composition is also likely.

Given the short‐term period of this study, it is difficult to propose specific fire management recommendations for the LNP. This is supported by recent considerations for the neighboring park, KNP in which even with over 50 years of fire management several challenges remain (van Wilgen, Govender, Smit, & MacFadyen, 2014). In fact, these authors refer that there is a need to continually improve understanding of the effects of fire, and to develop frameworks for assessing the impacts of fire together with other ecosystem drivers that interact strongly with fire to influence the attainment of ecological objectives.

In essence, our main recommendation for the LNP is to coordinate fire management activities with the neighboring KNP in establishing a harmonized management system that benefits both parks. In the short‐term and based on field observations more than on our results, we recommend the following actions: (a) regular annual burning of firebreaks for safety and security around infrastructures and touristic areas; and (b) regular burning for specific ecological reasons (i.e., reduction of bush thickening), protection of fire sensitive habitats (riparian areas) and removal of moribund material to keep areas open and encourage use by game (e.g., Nwambia Sandveld landscape).

A fire management system for the area should take into consideration local people's knowledge and perceptions about fire. In this sense, community‐based fire management in the LNP must focus on awareness the positive and negative effects of fires and, recover traditional practices.

Finally, given the observed changes in grass and woody species composition according to FF, we strongly recommend the establishment of a long‐term monitoring system (e.g., permanent sample plots). This would allow a systematic and consistent assessment of the role of fire on savanna ecosystems in LNP, contributing to adaptive management action.

## CONFLICT OF INTEREST

None declared.

## AUTHORS’ CONTRIBUTION

All authors have worked interactively to produce the submitted version of the article, each having different roles. NR and NG conceived the study, acquired the funding to implement it and coordinated data analysis. NR had a major role in coordinating the article writing and submission. GR was responsible for fire data analysis and wrote the fire component of the article. VM, RB, SNL and AC were involved in data collection and analysis as well as critically reviewing the article. AP and DM participated in data collection and analysis. All authors approved the final version of the paper.

## Data Availability

Data supporting the results of this paper will be made publicly available after publication through the DRYAD public repository (Provisional DOI: https://doi.org/10.5061/dryad.jn013r2).
